# Sodium-Glucose Cotransporter-2 Inhibitors and Nephritis Among Patients With Systemic Lupus Erythematosus

**DOI:** 10.1001/jamanetworkopen.2024.16578

**Published:** 2024-06-12

**Authors:** Fu-Shun Yen, Shiow-Ing Wang, Chih-Cheng Hsu, Chii-Min Hwu, James Cheng-Chung Wei

**Affiliations:** 1 Dr Yen’s Clinic, Taoyuan, Taiwan; 2 Center for Health Data Science, Department of Medical Research, Chung Shan Medical University Hospital, Taichung, Taiwan; 3 Institute of Medicine, Chung Shan Medical University, Taichung, Taiwan; 4 Institute of Population Health Sciences, National Health Research Institutes, Miaoli County, Taiwan; 5 Department of Health Services Administration, China Medical University, Taichung, Taiwan; 6 Department of Family Medicine, Min-Sheng General Hospital, Taoyuan, Taiwan; 7 National Center for Geriatrics and Welfare Research, National Health Research Institutes, Yunlin County, Taiwan; 8 Section of Endocrinology and Metabolism, Department of Medicine, Taipei Veterans General Hospital, Taipei, Taiwan; 9 Department of Medicine, National Yang-Ming Chiao Tung University School of Medicine, Taipei, Taiwan; 10 Department of Allergy, Immunology & Rheumatology, Chung Shan Medical University Hospital, Taichung City, Taiwan; 11 Graduate Institute of Integrated Medicine, China Medical University, Taichung, Taiwan

## Abstract

**Importance:**

Lupus nephritis is a major complication of systemic lupus erythematosus (SLE). Randomized clinical trials have shown nephroprotective and cardioprotective effects of sodium-glucose cotransporter-2 inhibitors (SGLT2is).

**Objective:**

To investigate whether the use of SGLT2is is associated with the onset and progression of lupus nephritis and other kidney and cardiac outcomes in patients with SLE and type 2 diabetes.

**Design, Setting, and Participants:**

This multicenter cohort study used the US Collaborative Network of the TriNetX clinical data platform to identify patients with SLE and type 2 diabetes from January 1, 2015, to December 31, 2022. Data collection and analysis were conducted in September 2023.

**Exposures:**

Individuals were categorized into 2 groups by SGLT2i use or nonuse with 1:1 propensity score matching.

**Main Outcomes and Measures:**

The Kaplan-Meier method and Cox proportional hazards regression models were used to calculate the 5-year adjusted hazard ratios (AHRs) of lupus nephritis, dialysis, kidney transplant, heart failure, and mortality for the 2 groups.

**Results:**

From 31 790 eligible participants, 1775 matched pairs of SGLT2i users and nonusers (N = 3550) were selected based on propensity scores. The mean (SD) age of matched participants was 56.8 (11.6) years, and 3012 (84.8%) were women. SGLT2i users had a significantly lower risk of lupus nephritis (AHR, 0.55; 95% CI, 0.40-0.77), dialysis (AHR, 0.29; 95% CI, 0.17-0.48), kidney transplant (AHR, 0.14; 95% CI, 0.03-0.62), heart failure (AHR, 0.65; 95% CI, 0.53-0.78), and all-cause mortality (AHR, 0.35; 95% CI, 0.26-0.47) than SGLT2i nonusers.

**Conclusions and Relevance:**

In this cohort study of patients with SLE and type 2 diabetes, SGLT2i users had a significantly lower risk of lupus nephritis, dialysis, kidney transplant, heart failure, and all-cause mortality than nonusers. The findings suggest that SGLT2is may provide some nephroprotective and cardioprotective benefits.

## Key Points

**Question:**

Is use of sodium-glucose cotransporter-2 inhibitors (SGLT2is) associated with onset and progression of lupus nephritis and other kidney and cardiac outcomes in patients with systemic lupus erythematosus (SLE) and type 2 diabetes?

**Findings:**

In this multicenter cohort study of 1775 matched pairs of SGLT2i users and nonusers with SLE and type 2 diabetes, use of SGLT2is was associated with significantly reduced risk of lupus nephritis, dialysis, kidney transplant, heart failure, and all-cause mortality compared with no SGLT2i use.

**Meaning:**

In this study, use of SGLT2is was associated with improved kidney and cardiac outcomes in patients with SLE and type 2 diabetes.

## Teaser

This cohort study investigates whether sodium-glucose cotransporter-2 inhibitor (SGLT2i) use is associated with lupus nephritis and other kidney and cardiac adverse outcomes in patients with systemic lupus erythematosus and type 2 diabetes.

## Introduction

Systemic lupus erythematosus (SLE) is a relapsing and life-threatening autoimmune disease that occurs when the body’s immune system has lost tolerance to its nuclear antigens.^1^ Patients with SLE have approximately 2.2-times higher mortality rates than individuals without SLE.^2^ Lupus nephritis is the most common and severe complication of SLE. Approximately 40% to 60% of patients with SLE have lupus nephritis, and most cases occur within 5 years of SLE diagnosis.^3^ Despite significant advances in the understanding and treatment of lupus nephritis in recent years, treatment outcomes for lupus nephritis remain unsatisfactory and lupus nephritis remains a major cause of morbidity and mortality in patients with SLE.^3^ Approximately 5% to 30% of patients with lupus nephritis may progress to kidney failure within 10 years.^4^ Patients who have SLE with kidney involvement have a 1.9-times higher mortality rate than those without kidney involvement.^5^ Moreover, patients with SLE and kidney disease are more likely to develop other organ-related diseases, such as heart disease, and have a lower quality of life.^4^

While SLE and type 2 diabetes do not commonly occur together, a patient with SLE and type 2 diabetes may have greater risk of poor outcomes because both conditions are associated with kidney and cardiovascular disease.^6^ Sodium-glucose cotransporter-2 inhibitors (SGLT2is) that lower blood glucose levels by inhibiting approximately 90% of glucose reabsorption in the proximal kidney tubule and increasing glycosuria were approved by the US Food and Drug Administration for treating type 2 diabetes in 2012.^7^ A meta-analysis of randomized clinical trials (RCTs) found that SGLT2is were associated with nephroprotective and cardioprotective effects, possibly mediated by reducing intraglomerular pressure.^8^ Two studies showed that SGLT2is significantly reduced proteinuria in patients (n = 5 and n = 9) with lupus nephritis.^9,10^ Zhao et al^10^ performed a study showing that empagliflozin could restore kidney SGLT2 overexpression, decrease mammalian target of rapamycin complex 1 (mTORC1) activity to attenuate inflammation, enhance autophagy, and alleviate podocyte damage in patients with lupus nephritis and in lupus-prone mice. Because patients with SLE and lupus nephritis were excluded from the cardiovascular and kidney RCTs due to their potential need for acute immunosuppression,^8^ we conducted this cohort study to investigate whether SGLT2i use might be associated with the onset and progression of lupus nephritis and other kidney and cardiac outcomes in patients with SLE and type 2 diabetes.

## Methods

### Study Design and Data Source

This retrospective cohort study used data aggregated from TriNetX, the world’s largest and most extensive clinical data and evidence ecosystem within the life sciences and health care domain. TriNetX encompasses deidentified electronic health records of over 250 million individuals sourced from more than 120 global health care organizations. TriNetX uses a standardized framework to ensure data quality that encompasses 3 primary categories of quality metrics: conformance, completeness, and plausibility.^11^ TriNetX has been used for executing numerous high-quality studies.^12,13^ The TriNetX platform adheres to the Health Insurance Portability and Accountability Act and the General Data Protection Regulation. The Western Institutional Review Board granted TriNetX a waiver of informed consent because it solely aggregates counts and statistical summaries of deidentified information. Furthermore, the use of TriNetX for this study received approval from the institutional review board of Chung Shan Medical University Hospital. The reporting of this study followed the Strengthening the Reporting of Observational Studies in Epidemiology (STROBE) reporting guideline.

We conducted data collection and analysis in September 2023 using the US Collaborative Network, a subnetwork of the TriNetX platform, for the relevant analyses. This network comprised 59 health care organizations. In line with our study’s objectives, we limited the study period to encompass dates between January 1, 2015, and December 31, 2022.

### Study Participants

Individuals eligible for this study were those diagnosed with SLE using *International Statistical Classification of Diseases, Tenth Revision, Clinical Modification (ICD-10-CM)* code M32 and with comorbid type 2 diabetes using codes E08 to E13, excluding E10. The definitions of SLE and type 2 diabetes are based on *International Statistical Classification of Diseases and Related Health Problems, Tenth Revision (ICD-10)* codes from outpatient or inpatient records. The algorithm of using *ICD-10-CM* and *ICD-10* codes to define these 2 diseases has been validated by previous studies.^14,15^ We excluded individuals aged younger than 20 years and those who had ever been diagnosed with type 1 diabetes as defined by *ICD-10-CM* code E10.

We subsequently categorized these individuals into 2 groups, distinguishing them by SGLT2i use as determined by the Anatomical Therapeutic Chemical code A10BK. SGLT2i users were identified as individuals who were prescribed an SGLT2i at least once during the study period. For SGLT2i users, the index date was set as the date of the first prescription. SGLT2i nonusers were defined as individuals with no recorded prescription of an SGLT2i in their electronic medical records at any point. For SGLT2i nonusers, the index date was established as the date of initial diagnosis of both SLE and type 2 diabetes. Patients in both cohorts were excluded if they had experienced any of the specified outcomes of interest (lupus nephritis, dialysis, kidney transplant, heart failure, or death) before or on the index date.

Race was ascertained by self-report and included in the analysis for generalizability of the findings. Categories were American Indian or Alaska Native, Asian, Black or African American, Native Hawaiian or Other Pacific Islander, White, other (included as a category in TriNetX and not broken down further), and unknown.

### Outcomes and Covariates

The adverse outcomes under investigation included lupus nephritis (as defined by *ICD-10-CM* codes M32.14-M32.15),^16^ dialysis (*ICD-10-CM* codes Z49.31, Z49.32, or Z99.2 or *Current Procedural Terminology* [*CPT*] code 1012740),^17^ kidney transplant (indicated by *ICD-10-CM* codes Z94.0 or *CPT* code 1008098, 0TY0, or 0TY1), heart failure (*ICD-10-CM* code I50),^18^ and all-cause mortality. The present study included the following covariate factors, assessed within 1 year before the index date to mitigate potential confounding effects: demographic variables, lifestyle factors, medical service utilization, diabetes severity, comorbidities, medications, and laboratory results.

### Statistical Analyses

To mitigate the influence of confounding factors, we used TriNetX’s built-in capability to generate propensity scores and 1:1 matching using greedy nearest neighbor matching. A caliper of 0.1 pooled SDs of the 2 groups was used during matching for variables. We assessed the comparability between the 2 groups before and after matching using standardized mean differences (SMDs). An SMD less than 0.1 indicated that the cohorts were well balanced.

We used Kaplan-Meier analysis to estimate the probability of survival free of the respective outcomes. The hazard ratios (HRs), their associated 95% CIs, and the test for proportionality were computed using the survival package in R, version 3.2.3 (R Project for Statistical Computing). The log-rank test results determined whether there were differences in survival curves between the cohorts and were conducted within the TriNetX platform.

We conducted several subgroup analyses to examine differences between groups defined by their initial diabetes management and severity based on glycated hemoglobin (HbA_1c_) and creatinine levels, estimated glomerular filtration rate (eGFR), and the presence or absence of chronic kidney disease or related complications. This was done to assess whether the association of SGLT2is with the onset of lupus nephritis varies under different conditions. We also conducted several sensitivity analyses to test the robustness of our results. First, we performed a competing risk analysis with mortality as the competing event. Second, we conducted an active comparator analysis to evaluate outcomes between SGLT2i and sulfonylurea users and between SGLT2i and dipeptidyl peptidase-4 (DPP-4) inhibitor users among patients with SLE. Third, we compared outcome risk between patients with at least 2 SGLT2i prescriptions and those without an SGLT2i prescription. Two-sided *P* < .05 was considered significant.

## Results

### Characteristics of Study Participants

**Figure 1.**
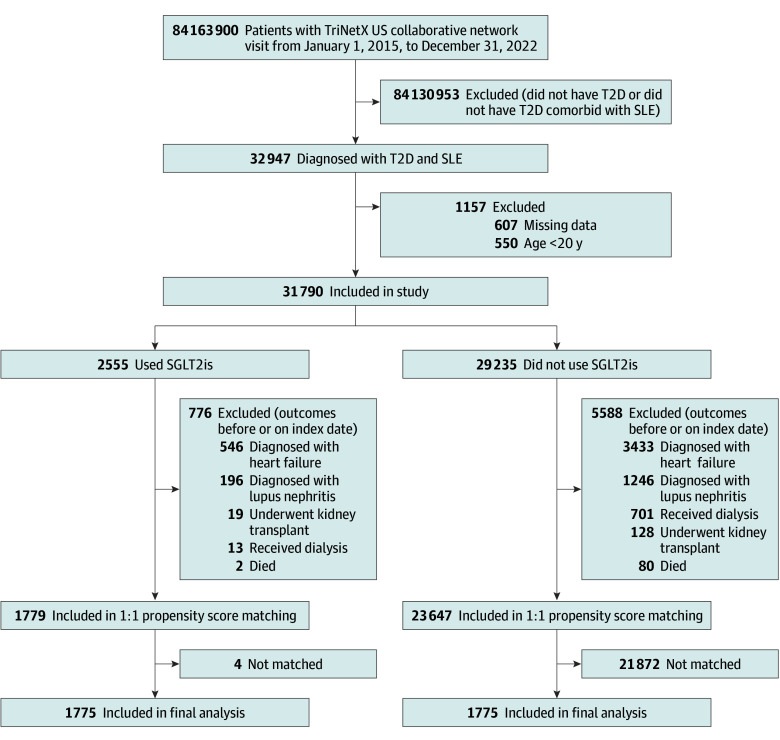
Flowchart of the Selection Process SGLT2i indicates sodium-glucose cotransporter-2 inhibitor; SLE, systemic lupus erythematosus; T2D, type 2 diabetes.

This study included 31 790 eligible participants initially. Following propensity score matching, 1775 patients were categorized as SGLT2i users, with an equal number in the SGLT2i nonuser group. The selection process is depicted in Figure 1.

The study participants’ baseline characteristics both before and after matching are displayed in Table 1. Before the matching process, disparities existed between the 2 groups regarding diabetes severity, comorbidities, medication use, and laboratory data. After matching, these discrepancies were minimal and fell within an acceptable range, with SMDs of less than 0.1 for the matched variables. The mean (SD) age of the 3550 matched participants was 56.8 (11.6) years; 480 (13.5%) were men, 3012 (84.8%) were women, and sex was unknown for 58 (1.6%). Twenty patients (0.6%) were American Indian or Alaska Native, 92 (2.6%) were Asian, 905 (25.5%) were Black or African American, 20 (0.6%) were Native Hawaiian or Other Pacific Islander, 1982 (55.8%) were White, 121 (4.4%) were other race and ethnicity, and 413 (11.6%) had unknown race and ethnicity (if there were 10 or fewer participants in matched groups within a category, the count is reported as 10).

**Table 1.** Baseline Characteristics of Study Participants Before and After Matching

Variable	Participants^a^					
	Before PSM			After PSM^b^		
	SGLT2i users (n = 1779)	SGLT2i nonusers (n = 23 647)	SMD	SGLT2i users (n = 1775)	SGLT2i nonusers (n = 1775)	SMD
Age at index, mean (SD), y	56.8 (11.2)	56.9 (13.4)	0.010	56.8 (11.2)	56.8 (12.1)	0.001
Sex						
Female	1517 (85.3)	19 790 (83.7)	0.044	1514 (85.3)	1498 (84.4)	0.025
Male	226 (12.7)	2843 (12.0)	0.021	225 (12.7)	255 (14.4)	0.049
Unknown	36 (2.0)	1014 (4.3)	0.130	36 (2.0)	22 (1.2)	0.062
Race and ethnicity						
American Indian or Alaska Native	10 (0.6)	167 (0.7)	0.018	10 (0.6)	10 (0.6)	0.000
Asian	50 (2.8)	582 (2.5)	0.022	49 (2.8)	43 (2.4)	0.021
Black or African American	457 (25.7)	6196 (26.2)	0.012	456 (25.7)	449 (25.3)	0.009
Native Hawaiian or Other Pacific Islander	10 (0.6)	115 (0.5)	0.010	10 (0.6)	10 (0.6)	0.000
White	974 (54.8)	11 967 (50.6)	0.083	973 (54.8)	1009 (56.8)	0.041
Other^c^	66 (3.7)	938 (4.0)	0.013	65 (3.7)	56 (3.2)	0.028
Unknown	213 (12.0)	3682 (15.6)	0.105	213 (12.0)	200 (11.3)	0.023
Potential health hazards related to socioeconomic and psychosocial circumstances	39 (2.2)	353 (1.5)	0.052	38 (2.1)	30 (1.7)	0.033
Lifestyle						
Nicotine dependence	140 (7.9)	1925 (8.1)	0.010	140 (7.9)	114 (6.4)	0.057
Personal history of nicotine dependence	147 (8.3)	1720 (7.3)	0.037	147 (8.3)	127 (7.2)	0.042
Tobacco use	34 (1.9)	520 (2.2)	0.020	34 (1.9)	36 (2.0)	0.008
Alcohol-related disorders	10 (0.6)	278 (1.2)	0.066	10 (0.6)	10 (0.6)	0.000
Alcoholic liver disease	10 (0.6)	80 (0.3)	0.033	10 (0.6)	10 (0.6)	0.000
Medical services utilization						
Office or other outpatient	1005 (56.5)	10 716 (45.3)	0.225	1001 (56.4)	923 (52.0)	0.088
Emergency department	369 (20.7)	5014 (21.2)	0.011	368 (20.7)	350 (19.7)	0.025
Hospital inpatient	132 (7.4)	2210 (9.3)	0.070	131 (7.4)	116 (6.5)	0.033
Preventive medicine	138 (7.8)	1018 (4.3)	0.145	138 (7.8)	101 (5.7)	0.083
Type 2 diabetes severity						
Without complications	982 (55.2)	6062 (25.6)	0.632	978 (55.1)	971 (54.7)	0.008
Neurological complications	204 (11.5)	874 (3.7)	0.297	201 (11.3)	228 (12.8)	0.047
Kidney complications	138 (7.8)	864 (3.7)	0.178	137 (7.7)	141 (7.9)	0.008
Ophthalmic complications	54 (3.0)	309 (1.3)	0.119	53 (3.0)	54 (3.0)	0.003
Circulatory complications	52 (2.9)	297 (1.3)	0.117	52 (2.9)	60 (3.4)	0.026
Hyperosmolarity	19 (1.1)	69 (0.3)	0.095	19 (1.1)	10 (0.6)	0.056
Ketoacidosis	10 (0.6)	21 (0.1)	0.083	10 (0.6)	10 (0.6)	0.000
Other specified complications^d^	487 (27.4)	1665 (7.0)	0.559	483 (27.2)	476 (26.8)	0.009
Unspecified complications	111 (6.2)	406 (1.7)	0.233	107 (6.0)	99 (5.6)	0.019
Comorbidities						
Hypertensive diseases	927 (52.1)	9928 (42.0)	0.204	923 (52.0)	963 (54.3)	0.045
Disorders of lipoprotein metabolism and other lipidemia	775 (43.6)	6299 (26.6)	0.360	773 (43.5)	687 (38.7)	0.099
Overweight or obesity	482 (27.1)	4247 (18.0)	0.220	481 (27.1)	459 (25.9)	0.028
Chronic lower respiratory diseases	354 (19.9)	4173 (17.6)	0.058	353 (19.9)	357 (20.1)	0.006
Ischemic heart diseases	192 (10.8)	2433 (10.3)	0.016	191 (10.8)	175 (9.9)	0.030
Liver diseases	191 (10.7)	1934 (8.2)	0.087	189 (10.6)	190 (10.7)	0.002
Chronic kidney disease	151 (8.5)	2602 (11.0)	0.085	150 (8.5)	211 (11.9)	0.114
Diseases of arteries, arterioles, and capillaries	139 (7.8)	2323 (9.8)	0.071	137 (7.7)	199 (11.2)	0.120
Cerebrovascular diseases	93 (5.2)	1456 (6.2)	0.040	92 (5.2)	118 (6.6)	0.062
Rheumatoid arthritis						
With rheumatoid factor	42 (2.4)	429 (1.8)	0.038	42 (2.4)	29 (1.6)	0.052
Without rheumatoid factor	183 (10.3)	2056 (8.7)	0.054	182 (10.3)	178 (10.0)	0.007
Medications						
Insulins and analogues	504 (28.3)	3200 (13.5)	0.370	502 (28.3)	393 (22.1)	0.142
Metformin	642 (36.1)	3232 (13.7)	0.537	638 (35.9)	435 (24.5)	0.251
Glucagon-like peptide-1 analogues	294 (16.5)	599 (2.5)	0.491	291 (16.4)	79 (4.5)	0.399
Dipeptidyl peptidase-4 inhibitors	208 (11.7)	525 (2.2)	0.379	208 (11.7)	86 (4.8)	0.251
Thiazolidinediones	47 (2.6)	129 (0.5)	0.168	46 (2.6)	15 (0.8)	0.135
Sulfonylureas	247 (13.9)	905 (3.8)	0.360	246 (13.9)	128 (7.2)	0.218
HMG-CoA reductase inhibitors	628 (35.3)	4781 (20.2)	0.342	625 (35.2)	482 (27.2)	0.175
α-Adrenoreceptor antagonists	25 (1.4)	211 (0.9)	0.048	25 (1.4)	18 (1.0)	0.036
ARBs						
Alone	304 (17.1)	2436 (10.3)	0.198	300 (16.9)	293 (16.5)	0.011
Combined with other drugs	10 (0.6)	36 (0.2)	0.069	10 (0.6)	10 (0.6)	0.000
Diuretics	466 (26.2)	5069 (21.4)	0.112	463 (26.1)	475 (26.8)	0.015
β-Blocking agents	425 (23.9)	4951 (20.9)	0.071	423 (23.8)	420 (23.7)	0.004
Calcium channel blockers	327 (18.4)	3795 (16.0)	0.062	325 (18.3)	336 (18.9)	0.016
ACE inhibitors only	329 (18.5)	2770 (11.7)	0.190	328 (18.5)	271 (15.3)	0.086
NSAIDs and antirheumatic products	539 (30.3)	5833 (24.7)	0.126	537 (30.3)	524 (29.5)	0.016
Aspirin	250 (14.1)	3075 (13.0)	0.031	248 (14.0)	252 (14.2)	0.006
Corticosteroids for systemic use	714 (40.1)	8719 (36.9)	0.067	712 (40.1)	722 (40.7)	0.011
Immunosuppressants	244 (13.7)	2774 (11.7)	0.060	242 (13.6)	218 (12.3)	0.040
Hydroxychloroquine	311 (17.5)	4236 (17.9)	0.011	311 (17.5)	324 (18.3)	0.019
Antineoplastic agents	188 (10.6)	1969 (8.3)	0.077	188 (10.6)	168 (9.5)	0.038
Laboratory results						
Hemoglobin A_1c_						
<7%	347 (19.5)	5289 (22.4)	0.070	345 (19.4)	526 (29.6)	0.239
≥9%	328 (18.4)	689 (2.9)	0.519	326 (18.4)	113 (6.4)	0.371
Serum, plasma, or blood creatinine level ≥1.5 mg/dL	77 (4.3)	1938 (8.2)	0.160	77 (4.3)	118 (6.6)	0.102
eGFR, mL/min/1.73m^2^^e^						
<30	83 (4.7)	1747 (7.4)	0.115	83 (4.7)	71 (4.0)	0.033
30 to <60	360 (20.2)	4180 (17.7)	0.065	359 (20.2)	349 (19.7)	0.014
60 to <90	729 (41.0)	7470 (31.6)	0.196	725 (40.8)	719 (40.5)	0.007
≥90	528 (29.7)	5689 (24.1)	0.127	525 (29.6)	512 (28.8)	0.016
Albumin-creatinine ratio in urine ≥300 units	10 (0.6)	59 (0.3)	0.049	10 (0.6)	10 (0.6)	0.000

Abbreviations: ACE, angiotensin-converting enzyme; ARB, angiotensin II receptor blocker; eGFR, estimated glomerular filtration rate; HMG-CoA, 3-hydroxy-3-methylglutaryl coenzyme A; NSAID, nonsteroidal anti-inflammatory drug; PSM, propensity score matching; SGLT2i, sodium-glucose cotransporter-2 inhibitor; SMD, standardized mean difference.

SI conversion factors: To convert percentage of total hemoglobin to proportion of total hemoglobin, multiply by 0.01; creatinine to μmol/L, multiply by 88.4.

^a^ Data are presented as number (percentage) of participants unless otherwise indicated. If there were 10 or fewer patients, the count is shown as 10.

^b^ Propensity score matching was performed on age at index, sex, race, socioeconomic status, lifestyle, medical service utilization, diabetes severity, ARBs, and eGFR.

^c^ Included as a category in the TriNetX platform and not broken down further.

^d^ Issues such as skin complications, dental problems, digestive problems, or other organ-specific conditions directly related to diabetes but specified separately due to their unique nature.

^e^ Estimated by creatinine-based formula (modification of diet in kidney disease).

### Outcomes

**Figure 2.**
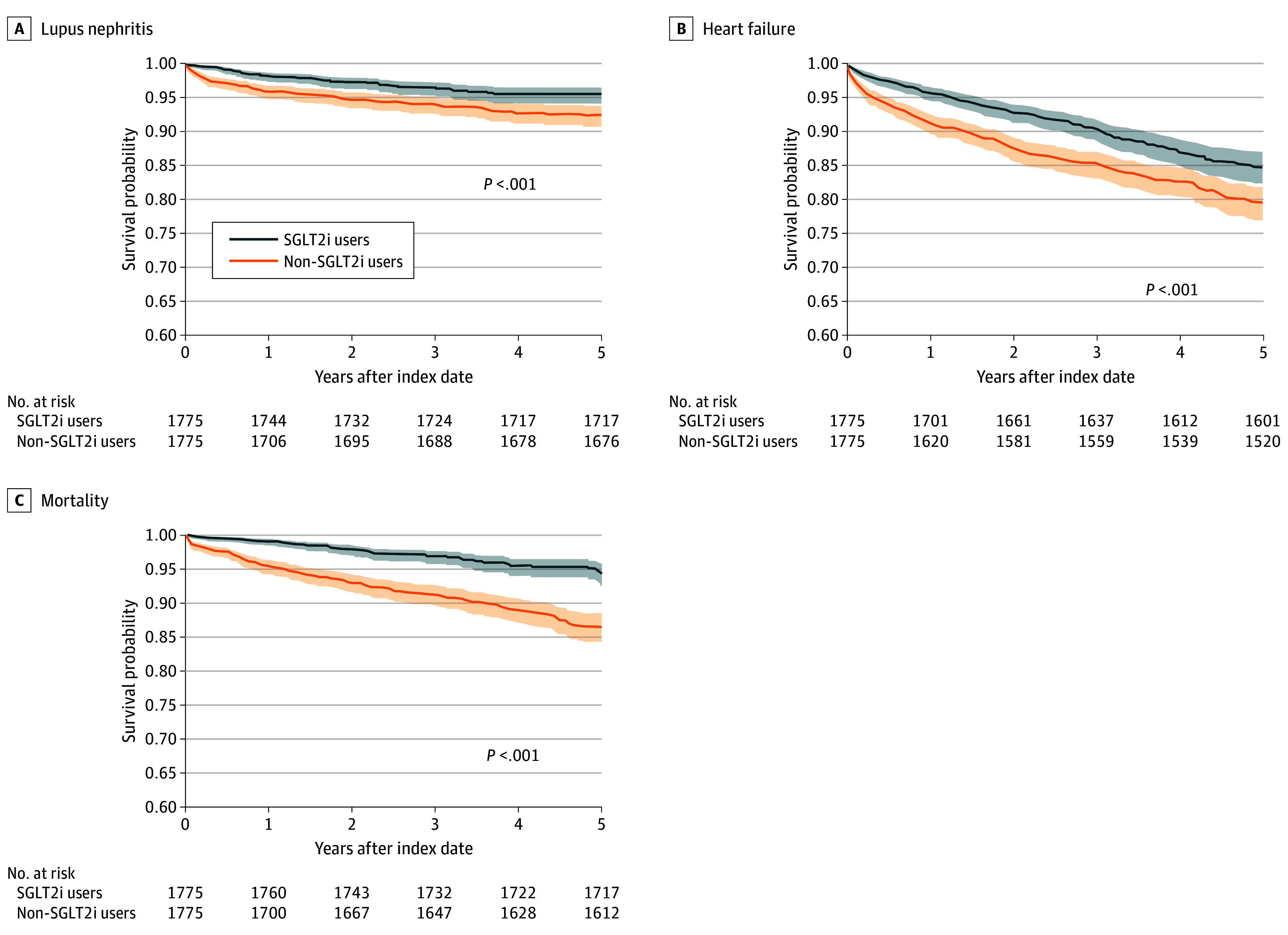
Event-Free Survival Probabilities SGLT2i indicates sodium-glucose cotransporter-2 inhibitor.

Table 2 presents the number of patients with outcomes in both cohorts accompanied by the 5-year adjusted HRs (AHRs) for the incidence of these outcomes in SGLT2i users compared with SGLT2i nonusers. SGLT2i users had a lower risk of lupus nephritis (AHR, 0.55; 95% CI, 0.40-0.77) (Figure 2A), dialysis (AHR, 0.29; 95% CI, 0.17-0.48), kidney transplant (AHR, 0.14; 95% CI, 0.03-0.62), heart failure (AHR, 0.65; 95% CI, 0.53-0.78) (Figure 2B), and all-cause mortality (AHR, 0.35; 95% CI, 0.26-0.47) (Figure 2C) than SGLT2i nonusers. Even when accounting for various variables (eTable 1 in Supplement 1) or considering different follow-up durations (eTable 2 in Supplement 1), we consistently observed similar findings. The competing risk analyses using death as a competing risk also showed that SGLT2i use was associated with lower risk of lupus nephritis (AHR, 0.43; 95% CI, 0.35-0.54), dialysis (AHR, 0.34; 95% CI, 0.26-0.44), kidney transplant (AHR, 0.33; 95% CI, 0.25-0.45), heart failure (AHR, 0.57; 95% CI, 0.48-0.68), and all-cause mortality (AHR, 0.35; 95% CI, 0.26-0.47) compared with no use of SGLT2is (eTable 3 in Supplement 1).

**Table 2.** Risk of Outcomes at 1 Day to 5 Years

Outcome	Patients, No. (%)^a^		Adjusted hazard ratio (95% CI)^b^
	SGLT2i users (n = 1775)	SGLT2i nonusers (n = 1775)	
Lupus nephritis	58 (3.27)	99 (5.58)	0.55 (0.40-0.77)
Dialysis	19 (1.07)	64 (3.60)	0.29 (0.17-0.48)
Kidney transplant	10 (0.56)	14 (0.79)	0.14 (0.03-0.62)
Heart failure	174 (9.80)	255 (14.37)	0.65 (0.53-0.78)
All-cause mortality	58 (3.27)	163 (9.18)	0.35 (0.26-0.47)

Abbreviation: SGLT2i, sodium-glucose cotransporter-2 inhibitor.

^a^ Propensity score matching was performed on age at index, sex, race, socioeconomic status, lifestyle, medical service utilization, diabetes severity, angiotensin II receptor blockers, and estimated glomerular filtration rate. If there were 10 or fewer patients, the count is shown as 10.

^b^ Adjusted for the variables listed in Table 1.

### Subgroup Analyses

We further investigated the risk of outcomes by dividing the patient population into subgroups based on diabetes management status as determined by HbA_1c_ levels (eTable 4 in Supplement 1). Among patients with HbA_1c_ levels below 7% (to convert percentage of total hemoglobin to proportion of total hemoglobin, multiply by 0.01), SGLT2i users had a lower risk of lupus nephritis, heart failure, and all-cause mortality than SGLT2i nonusers. However, for individuals with HbA_1c_ levels above 7%, SGLT2i users with HbA_1c_ levels either exceeding or below 9% had a lower risk of lupus nephritis, dialysis, heart failure, and all-cause mortality than SGLT2i nonusers with those HbA_1c_ levels (eTable 5 in Supplement 1).

Regardless of whether creatinine levels were greater or less than 1.5 mg/dL (to convert to μmol/L, multiply by 88.4), SGLT2i users consistently had lower risk of lupus nephritis, dialysis, heart failure, and all-cause mortality than SGLT2i nonusers (eTable 6 in Supplement 1). Among patients with eGFR less than 60 mL/min/1.73 m^2^, SGLT2i users had lower risk of lupus nephritis, dialysis, heart failure, and all-cause mortality than SGLT2i nonusers (eTable 7 in Supplement 1). For patients with eGFR 60 mL/min/1.73 m^2^ or greater, SGLT2i users had lower risk of lupus nephritis, heart failure, and all-cause mortality than SGLT2i nonusers.

**Figure 3.**
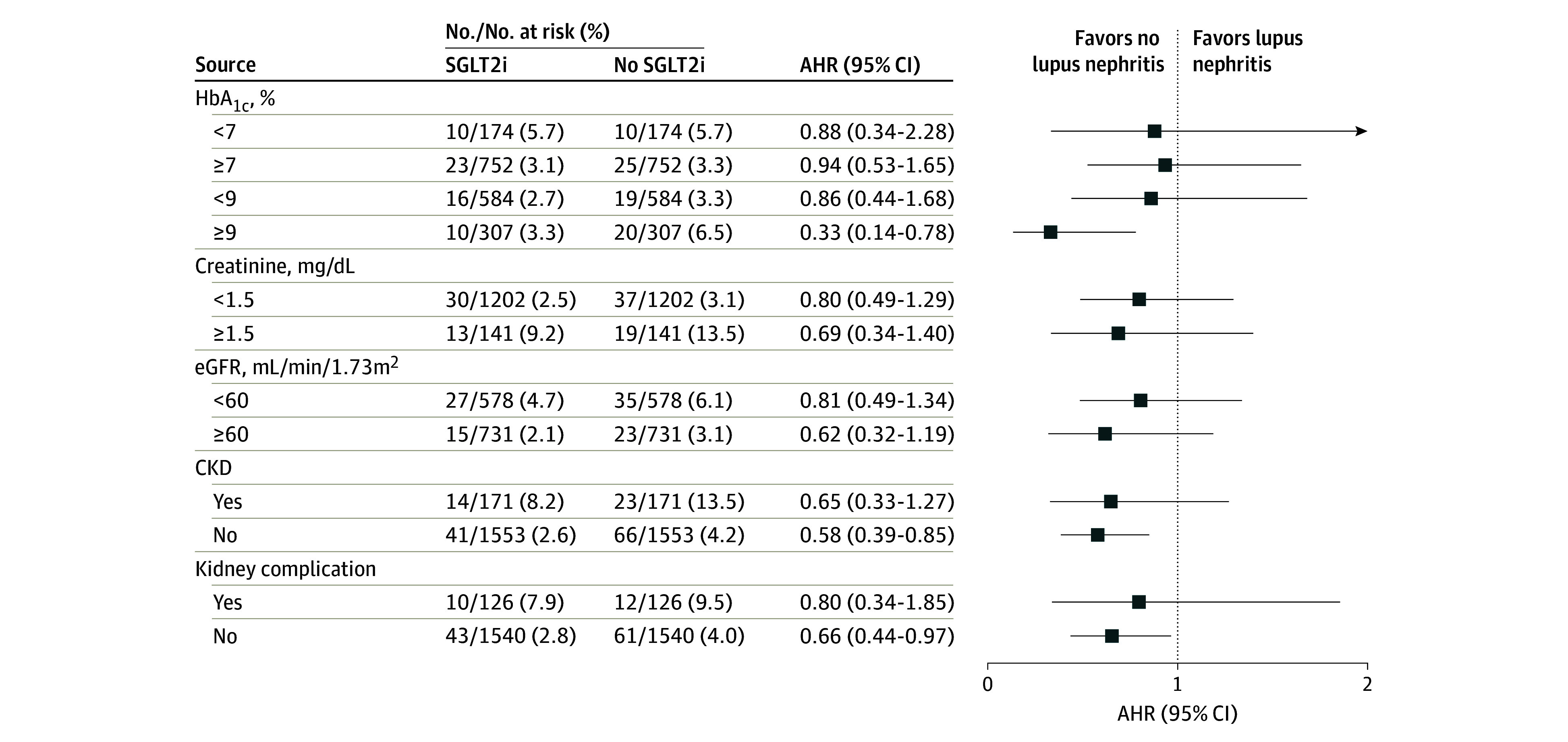
Subgroup Analyses of the Risk of Incident Lupus Nephritis by Sodium-Glucose Cotransporter-2 Inhibitor (SGLT2i) Use To convert percentage of total hemoglobin to proportion of total hemoglobin, multiply by 0.01; creatinine to μmol/L, multiply by 88.4. CKD indicates chronic kidney disease; eGFR, estimated glomerular filtration rate; HbA_1c_, glycated hemoglobin.

In patients with or without chronic kidney disease and kidney complications, SGLT2i users also had a lower risk of lupus nephritis, dialysis, heart failure, and all-cause mortality than SGLT2i nonusers (eTables 8 and 9 in Supplement 1). Figure 3 shows the results of all subgroup analyses for the risk of incident lupus nephritis.

### Sensitivity Analyses

In the matched cohorts, SGLT2i use was associated with a lower risk of lupus nephritis, dialysis, kidney transplant, heart failure, and all-cause mortality compared with use of sulfonylureas (eTable 10 in Supplement 1). Compared with the matched DPP-4 inhibitor users, SGLT2i users had a lower risk of dialysis, kidney transplant, heart failure, and all-cause mortality (eTable 11 in Supplement 1). Patients with at least 2 prescriptions of SGLT2is had a lower risk of lupus nephritis, dialysis, kidney transplant, heart failure, and all-cause mortality than SGLT2i nonusers (eTable 12 in Supplement 1).

## Discussion

This cohort study showed that SGLT2i use was associated with a significantly lower risk of incident lupus nephritis, dialysis, kidney transplant, heart failure, and all-cause mortality compared with SGLT2i nonuse in patients with SLE and type 2 diabetes. This finding was consistent in different variable-adjusted models. Our study suggests that SGLT2is may provide multiple benefits beyond glycemic control, including nephroprotection and cardioprotection in patients with both SLE and type 2 diabetes.

A phase I/II trial found increased glucose metabolism within immune cells of individuals with SLE.^19^ Taming this overreactive immune response through metabolic modulation has emerged as a potential therapeutic strategy for SLE.^19^ In an RCT, metformin demonstrated the capability to decrease the likelihood of lupus flare-ups.^20^ Results of RCTs have suggested SGLT2i use for the treatment of hyperglycemia and primary prevention of chronic kidney disease in patients with type 2 diabetes.^21,22,23^ SGLT2is may reduce the risk of diabetic kidney disease by increasing glycosuria and improving glomerular hyperfiltration.^7^ Since the development of lupus nephritis is also associated with increased intraglomerular pressure, SGLT2is may reduce the risk of lupus nephritis in patients with SLE.^8^ Two pilot studies showed that SGLT2i could significantly reduce proteinuria in patients with SLE and lupus nephritis.^9,10^ However, these studies had limited participant numbers (5 and 9 patients each). A preclinical study by Zhao and colleagues^10^ demonstrated that empagliflozin administration could reduce the levels of mouse anti–double stranded (ds) DNA immunoglobulin G (IgG)–specific antibodies, serum creatinine, and protein in urine; ameliorate glomerular and tubulointerstitial damage; increase the expression of synaptopodin and reverse podocyte injury caused by SGLT2 overexpression; alleviate podocyte apoptosis by attenuating inflammation; and increase autophagy by reducing mTORC1 activity in patients with lupus nephritis and lupus-prone mice. To our knowledge, our study is the first to report that SGLT2i use may be associated with decreased onset of lupus nephritis in patients with SLE and type 2 diabetes. Moreover, the results remained consistent when analyzed using different models of the variables and over a period of follow-up that ranged from 1 to 5 years.

There are possible explanations for SGLT2i use being associated with a lower risk of incident lupus nephritis. First, SGLT2is can increase natriuresis, which triggers the tubuloglomerular feedback mechanism.^7^ This mechanism results in vasoconstriction of the afferent arterioles, which attenuates intraglomerular hypertension, decreases glomerular shear stress, and reduces protein levels in urine. It also reduces tubular workload, oxygen demand, and injury.^8^ Second, SGLT2is may exert immunomodulatory effects by reducing dsDNA autoantibodies, decreasing immune complex deposition in kidney tissues, and attenuating kidney dysfunction and lupus nephritis development.^10^ Third, SGLT2is can increase ketone production and block lipopolysaccharide-induced and NLRP3-mediated inflammation. They also affect macrophage polarization through interactions with the mTOR and AMP-activated protein kinase pathways. Thus, they help decrease oxidative stress, reduce proinflammatory cytokines and inflammation, attenuate podocyte damage, and regulate endothelial dysfunction.^10,19,24^ Fourth, SGLT2is can inhibit apoptosis and the production of reactive oxygen species and attenuate glomerular atrophy, kidney fibrosis, and kidney dysfunction.^25,26^

A previous systematic review and meta-analysis of RCTs demonstrated that SGLT2i use was associated with slowing the progression of chronic kidney disease to kidney failure and kidney replacement therapy in people with or without diabetes.^27^ A prespecified analysis of the Dapagliflozin and Prevention of Adverse Outcomes in Chronic Kidney Disease Trial showed that dapagliflozin could significantly reduce the progression of chronic kidney disease in patients with IgA nephropathy.^28^ Uncontrolled lupus nephritis may worsen over time and lead to chronic kidney disease.^4,29^ Moreover, about 25% of patients with diabetes will develop chronic kidney disease.^30^ In our study, some patients had chronic kidney disease. However, SGLT2i use was associated with a significantly lower risk of dialysis and kidney transplant compared with no use of SGLT2i in patients with SLE and type 2 diabetes. Furthermore, the observed protective association remained consistent in patients with HbA_1c_ levels of 7% or higher, creatinine levels less than 1.5 mg/dL, and eGFR less than 60 mL/min/1.73 m^2^.

Patients with SLE have an increased risk of atherosclerosis and cardiovascular disease due to vascular inflammation.^1,29^ Cardiovascular disease is also a major complication and cause of death in patients with diabetes.^31^ A meta-analysis of RCTs showed that SGLT2i use was associated with preventing the development of heart failure and slowing its progression at various stages, benefiting people with and without diabetes.^32^ The present study showed that SGLT2i use was associated with a significantly lower risk of heart failure compared with SGLT2i nonuse in patients with SLE and type 2 diabetes. Furthermore, a consistent protective association was observed across different analytical models, over 1 to 5 years of follow-up, and in patients with HbA_1c_ levels of 7% or higher and normal or abnormal kidney function.

People with SLE have approximately double the mortality rate of those who do not have SLE.^2^ The numerous complications associated with diabetes also amplify the risk of death.^31^ The mortality risk is substantially higher for someone diagnosed with both SLE and type 2 diabetes compared with someone who has only 1 of these conditions.^6^ A systematic review and meta-analysis of RCTs demonstrated that SGLT2i use was associated with decreased risk of mortality for patients regardless of whether they had type 2 diabetes or chronic kidney disease.^27^ Our study showed that for patients with SLE and type 2 diabetes, SGLT2i use was associated with a significantly reduced risk of all-cause mortality compared with non-SGLT2i treatments. The reduced mortality observed in patients with SLE who used SGLT2i may be due to the association of use of this drug class with reduced incidence of lupus nephritis, dialysis, kidney transplant, and heart failure.

### Limitations

There are some limitations to this study. First, in attempting to match certain medications, HbA_1c_, and kidney function, we faced challenges in achieving optimal matching between the SGLT2i and control groups. However, we could successfully match diabetes complications and chronic kidney disease to balance the severity of diabetes and kidney disease between these 2 groups. Furthermore, we performed subgroup analyses based on different levels of HbA_1c_ and kidney function to see if results varied with different levels of kidney function and glycemic control. Second, we obtained these data for patients treated in hospitals with the possibility of missing or incomplete data on patients with mild or asymptomatic SLE or lupus nephritis treated in outpatient clinics, leading to selection bias or misclassification of data. However, this problem could affect both the SGLT2i and the control groups, potentially leading to an unbiased discrepancy between the 2 groups. The electronic medical record system in TriNetX also has some missing information, such as sex and race for some patients. Data on smoking habits were lacking, so we used diagnostic codes as a proxy for this missing information; however, there may be problems with coding completeness. Nevertheless, if a patient’s smoking history was recorded, the specificity should be high. Third, we determined the incidence of lupus nephritis using *ICD-10-CM* codes without pathologic confirmation by biopsy. However, a previous validation study showed that *ICD-10-CM* codes can accurately identify the occurrence of lupus nephritis.^16^ Fourth, certain risk factors may not be encompassed in the collected data; therefore, the propensity score matching method may not completely rectify potential imbalances in patient risk between the SGLT2i and control groups. Additionally, a retrospective cohort study often has unknown or unmeasured confounding factors even after adjusting for many important variables. Thus, the results indicate associations rather than causal relationships.

## Conclusions

This cohort study revealed that SGLT2i use in patients with SLE and type 2 diabetes was associated with a significantly reduced risk of lupus nephritis, dialysis, kidney transplant, heart failure, and all-cause mortality compared with SGLT2i nonuse. SGLT2is may provide some nephroprotective and cardioprotective benefits in patients with SLE and type 2 diabetes. However, these findings need to be confirmed by more rigorous, prospective RCTs.

## Notes

Supplement 1. **eTable 1.** Risk of Outcomes From 1 Day to 5 Years, Adjusted for Different Variables **eTable 2.** Risk of Outcomes by Follow-Up Duration **eTable 3.** Risk of Outcomes With the Competing Risk of Death **eTable 4.** Risk of Outcomes Stratified by HbA_1c_ Level Above or Below 7% **eTable 5.** Risk of Outcomes Stratified by HbA_1c_ Level Above or Below 9% **eTable 6.** Risk of Outcomes Stratified by Creatinine Level **eTable 7.** Risk of Outcomes Stratified by eGFR **eTable 8.** Risk of Outcomes Stratified by CKD **eTable 9.** Risk of Outcomes Stratified by Kidney Complications **eTable 10.** Risk of Outcomes Between Matched SGLT2i and Sulfonylurea Users Among Patients With Systemic Lupus Erythematosus **eTable 11.** Risk of Outcomes Between Matched SGLT2i and DPP-4i Users Among Patients With Systemic Lupus Erythematosus **eTable 12.** Risk of Outcomes With at Least 2 vs No SGLT2i Prescriptions

Supplement 2.
Data Sharing Statement

## References

[1] Rahman A, Isenberg DA. Systemic lupus erythematosus. N Engl J Med. 2008;358(9):929-939. doi:10.1056/NEJMra071297 18305268

[2] Chen YM, Lin CH, Chen HH, . Onset age affects mortality and renal outcome of female systemic lupus erythematosus patients: a nationwide population-based study in Taiwan. Rheumatology (Oxford). 2014;53(1):180-185. doi:10.1093/rheumatology/ket330 24136069

[3] Mohan C, Zhang T, Putterman C. Pathogenic cellular and molecular mediators in lupus nephritis. Nat Rev Nephrol. 2023;19(8):491-508. doi:10.1038/s41581-023-00722-z 37225921

[4] Mok CC, Teng YKO, Saxena R, Tanaka Y. Treatment of lupus nephritis: consensus, evidence and perspectives. Nat Rev Rheumatol. 2023;19(4):227-238. doi:10.1038/s41584-023-00925-5 36864291

[5] Mok CC, Kwok RC, Yip PS. Effect of renal disease on the standardized mortality ratio and life expectancy of patients with systemic lupus erythematosus. Arthritis Rheum. 2013;65(8):2154-2160. doi:10.1002/art.38006 23754671

[6] Cortes S, Chambers S, Jerónimo A, Isenberg D. Diabetes mellitus complicating systemic lupus erythematosus—analysis of the UCL lupus cohort and review of the literature. Lupus. 2008;17(11):977-980. doi:10.1177/0961203308091539 18852220

[7] Braunwald E. Gliflozins in the management of cardiovascular disease. N Engl J Med. 2022;386(21):2024-2034. doi:10.1056/NEJMra2115011 35613023

[8] McGuire DK, Shih WJ, Cosentino F, et al. Association of SGLT2 inhibitors with cardiovascular and kidney outcomes in patients with type 2 diabetes: a meta-analysis. JAMA Cardiol. 2021;6(2):148-158. doi:10.1001/jamacardio.2020.4511 33031522 PMC7542529

[9] Morales E, Galindo M. SGLT2 inhibitors in lupus nephropathy, a new therapeutic strategy for nephroprotection. Ann Rheum Dis. 2022;81:1337-1378. doi:10.1136/annrheumdis-2022-222512 35551062

[10] Zhao XY, Li SS, He YX, . SGLT2 inhibitors alleviated podocyte damage in lupus nephritis by decreasing inflammation and enhancing autophagy. Ann Rheum Dis. 2023;82(10):1328-1340. doi:10.1136/ard-2023-224242 37487609

[11] Kahn MG, Callahan TJ, Barnard J, . A harmonized data quality assessment terminology and framework for the secondary use of electronic health record data. EGEMS (Wash DC). 2016;4(1):1244. doi:10.13063/2327-9214.1244 27713905 PMC5051581

[12] Taquet M, Sillett R, Zhu L, . Neurological and psychiatric risk trajectories after SARS-CoV-2 infection: an analysis of 2-year retrospective cohort studies including 1 284 437 patients. Lancet Psychiatry. 2022;9(10):815-827. doi:10.1016/S2215-0366(22)00260-7 35987197 PMC9385200

[13] Paljarvi T, Forton J, Luciano S, Herttua K, Fazel S. Analysis of neuropsychiatric diagnoses after montelukast initiation. JAMA Netw Open. 2022;5(5):e2213643. doi:10.1001/jamanetworkopen.2022.13643 35608857 PMC9131741

[14] Barnado A, Carroll R, Denny JC, Crofford L. Using *ICD-10-CM* codes to identify patients with systemic lupus erythematosus in the electronic health record. *Arthritis Rheumatol*. 2018;70(suppl 9).

[15] Chi GC, Li X, Tartof SY, Slezak JM, Koebnick C, Lawrence JM. Validity of *ICD-10-CM* codes for determination of diabetes type for persons with youth-onset type 1 and type 2 diabetes. BMJ Open Diabetes Res Care. 2019;7(1):e000547. doi:10.1136/bmjdrc-2018-000547 30899525 PMC6398816

[16] Li T, Lee I, Jayakumar D, . Development and validation of lupus nephritis case definitions using United States Veterans Affairs electronic health records. Lupus. 2021;30(3):518-526. doi:10.1177/0961203320973267 33176569

[17] Grams ME, Plantinga LC, Hedgeman E, ; CDC CKD Surveillance Team. Validation of CKD and related conditions in existing data sets: a systematic review. Am J Kidney Dis. 2011;57(1):44-54. doi:10.1053/j.ajkd.2010.05.013 20692079 PMC2978782

[18] Ezekowitz JA, Bakal JA, Kaul P, Westerhout CM, Armstrong PW. Acute heart failure in the emergency department: short and long-term outcomes of elderly patients with heart failure. Eur J Heart Fail. 2008;10(3):308-314. doi:10.1016/j.ejheart.2008.01.014 18280788

[19] Wang H, Li T, Sun F, . Safety and efficacy of the SGLT2 inhibitor dapagliflozin in patients with systemic lupus erythematosus: a phase I/II trial. RMD Open. 2022;8(2):e002686. doi:10.1136/rmdopen-2022-002686 36288823 PMC9615980

[20] Sun F, Wang HJ, Liu Z, . Safety and efficacy of metformin in systemic lupus erythematosus: a multicentre, randomised, double-blind, placebo-controlled trial. Lancet Rheumatol. 2020;2(4):e210-e216. doi:10.1016/S2665-9913(20)30004-7 38268156

[21] Wanner C, Inzucchi SE, Lachin JM, ; EMPA-REG OUTCOME Investigators. Empagliflozin and progression of kidney disease in type 2 diabetes. N Engl J Med. 2016;375(4):323-334. doi:10.1056/NEJMoa1515920 27299675

[22] Neal B, Perkovic V, Mahaffey KW, ; CANVAS Program Collaborative Group. Canagliflozin and cardiovascular and renal events in type 2 diabetes. N Engl J Med. 2017;377(7):644-657. doi:10.1056/NEJMoa1611925 28605608

[23] Wiviott SD, Raz I, Bonaca MP, ; DECLARE–TIMI 58 Investigators. Dapagliflozin and cardiovascular outcomes in type 2 diabetes. N Engl J Med. 2019;380(4):347-357. doi:10.1056/NEJMoa1812389 30415602

[24] Dilliraj LN, Schiuma G, Lara D, . The evolution of ketosis: potential impact on clinical conditions. Nutrients. 2022;14(17):3613. doi:10.3390/nu14173613 36079870 PMC9459968

[25] Verma S, Jüni P, Mazer CD. Pump, pipes, and filter: do SGLT2 inhibitors cover it all? Lancet. 2019;393(10166):3-5. doi:10.1016/S0140-6736(18)32824-1 30424891

[26] Chang WT, Wu CC, Liao IC, . Dapagliflozin protects against doxorubicin-induced nephrotoxicity associated with nitric oxide pathway—a translational study. Free Radic Biol Med. 2023;208:103-111. doi:10.1016/j.freeradbiomed.2023.08.013 37549754

[27] Nuffield Department of Population Health Renal Studies Group; SGLT2 inhibitor Meta-Analysis Cardio-Renal Trialists’ Consortium. Impact of diabetes on the effects of sodium glucose co-transporter-2 inhibitors on kidney outcomes: collaborative meta-analysis of large placebo-controlled trials. Lancet. 2022;400(10365):1788-1801. doi:10.1016/S0140-6736(22)02074-8 36351458 PMC7613836

[28] Wheeler DC, Toto RD, Stefánsson BV, ; DAPA-CKD Trial Committees and Investigators. A pre-specified analysis of the DAPA-CKD trial demonstrates the effects of dapagliflozin on major adverse kidney events in patients with IgA nephropathy. Kidney Int. 2021;100(1):215-224. doi:10.1016/j.kint.2021.03.033 33878338

[29] Anders HJ, Saxena R, Zhao MH, Parodis I, Salmon JE, Mohan C. Lupus nephritis. Nat Rev Dis Primers. 2020;6(1):7. doi:10.1038/s41572-019-0141-9 31974366

[30] Wang JS, Yen FS, Lin KD, Shin SJ, Hsu YH, Hsu CC; Diabetes Kidney Disease Research Committee of the Diabetes Association of the Republic of China (Taiwan). Epidemiological characteristics of diabetic kidney disease in Taiwan. J Diabetes Investig. 2021;12(12):2112-2123. doi:10.1111/jdi.13668 34529360 PMC8668071

[31] ElSayed NA, Aleppo G, Aroda VR, ; on behalf of the American Diabetes Association. 10. Cardiovascular disease and risk management: standards of care in diabetes—2023. Diabetes Care. 2023;46(suppl 1):S158-S190. doi:10.2337/dc23-S010 36507632 PMC9810475

[32] Vaduganathan M, Docherty KF, Claggett BL, . SGLT-2 inhibitors in patients with heart failure: a comprehensive meta-analysis of 5 randomised controlled trials. Lancet. 2022;400(10354):757-767. doi:10.1016/S0140-6736(22)01429-5 36041474

