# Supplementary material for: Sodium-Glucose Cotransporter-2 Inhibitors and Nephritis Among Patients With Systemic Lupus Erythematosus
Source: JAMA Netw Open. 2024 Jun 12;7(6):e2416578. doi: 10.1001/jamanetworkopen.2024.16578 (PMC11170305; doi:10.1001/jamanetworkopen.2024.16578)
Supplement: Supplement 1. — eTable 1. Risk of Outcomes From 1 Day to 5 Years, Adjusted for Different Variables eTable 2. Risk of Outcomes by Follow-Up Duration eTable 3. Risk of Outcomes With the Competing Risk of Death eTable 4. Risk of Outcomes Stratified by HbA1c Level Above or Below 7% eTable 5. Risk of Outcomes Stratified by HbA1c Level Above or Below 9% eTable 6. Risk of Outcomes Stratified by Creatinine Level eTable 7. Risk of Outcomes Stratified by eGFR eTable 8. Risk of Outcomes Stratified by CKD eTable 9. Risk of Outcomes Stratified by Kidney Complications eTable 10. Risk of Outcomes Between Matched SGLT2i and Sulfonylurea Users Among Patients With Systemic Lupus Erythematosus eTable 11. Risk of Outcomes Between Matched SGLT2i and DPP-4i Users Among Patients With Systemic Lupus Erythematosus eTable 12. Risk of Outcomes With at Least 2 vs No SGLT2i Prescriptions [file jamanetwopen-e2416578-s001.pdf]

# Supplementary Online Content

Yen FS, Wang SI, Hsu CC, Hwu CM, Wei JCC. Sodium-glucose cotransporter-2 inhibitors and nephritis among patients with systemic lupus erythematosus. *JAMA Netw Open*. 2024;5(4):e214732. doi:10.1001/jamanetworkopen.2021.4732

**eTable 1.** Risk of Outcomes From 1 Day to 5 Years, Adjusted for Different Variables
**eTable 2.** Risk of Outcomes by Follow-Up Duration
**eTable 3.** Risk of Outcomes With the Competing Risk of Death
**eTable 4.** Risk of Outcomes Stratified by $HbA_{1c}$ Level Above or Below 7%
**eTable 5.** Risk of Outcomes Stratified by $HbA_{1c}$ Level Above or Below 9%
**eTable 6.** Risk of Outcomes Stratified by Creatinine Level
**eTable 7.** Risk of Outcomes Stratified by eGFR
**eTable 8.** Risk of Outcomes Stratified by CKD
**eTable 9.** Risk of Outcomes Stratified by Kidney Complications
**eTable 10.** Risk of Outcomes Between Matched SGLT2i and Sulfonylurea Users Among Patients With Systemic Lupus Erythematosus
**eTable 10.** Risk of Outcomes Between Matched SGLT2i and DPP-4i Users Among Patients With Systemic Lupus Erythematosus
**eTable 12.** Risk of Outcomes With at Least 2 vs No SGLT2i Prescriptions

This supplementary material has been provided by the authors to give readers additional information about their work.

© 2024 Yen FS et al. *JAMA Network Open*.

**eTable 1. Risk of outcomes (1 day to 5 years) adjusted different variables**

| Outcomes (SGLT2i users vs. Non-SGLT2i users) | Hazard ratio (95% CI) | | | | |
|---|---|---|---|---|---|
| | Model 1[a] | Model 2[b] | Model 3[c] | Model 4[d] | Model 5[e] |
| Lupus nephritis | **0.47 (0.34-0.58)** | **0.59 (0.42-0.81)** | **0.55 (0.40-0.76)** | 0.75 (0.53-1.05) | 0.92 (0.64-1.33) |
| Dialysis | **0.24 (0.15-0.38)** | **0.23 (0.14-0.37)** | **0.29 (0.17-0.48)** | **0.38 (0.22-0.64)** | **0.54 (0.31-0.94)** |
| Kidney transplant | **0.06 (0.02-0.26)** | **0.09 (0.02-0.38)** | **0.14 (0.03-0.62)** | **0.13 (0.03-0.58)** | 0.32 (0.06-1.57) |
| Heart failure | **0.64 (0.55-0.74)***  | **0.60 (0.50-0.72)** | **0.65 (0.53-0.78)** | **0.76 (0.62-0.93)** | **0.78 (0.64-0.95)** |
| All-cause mortality | **0.31 (0.24-0.40)** | **0.29 (0.22-0.39)** | **0.35 (0.26-0.47)** | **0.37 (0.27-0.50)** | **0.39 (0.29-0.53)** |

SGLT2i: Sodium-glucose co-transporter 2 inhibitors; CI: Confidence interval.

[a] Crude, before matching.

[b] Propensity score matching was performed on age, sex, race, and DM severity.

[c] Propensity score matching was performed on age at index, sex, race, social economic status, lifestyles, medical utilization, DM severity, angiotensin II receptor blockers (ARB), and estimated glomerular filtration rate (eGFR).

[d] Propensity score matching was performed on age at index, sex, race, social economic status, lifestyles, medical utilization, DM severity, comorbidities, angiotensin II receptor blockers (ARB), and estimated glomerular filtration rate (eGFR).

[e] Propensity score matching was performed on all listed characteristics.

* Proportionality <0.001.

© 2024 Yen FS et al. *JAMA Network Open*.

**eTable 2. Risk of outcomes different follow-up duration**

| Outcomes (SGLT2i users vs. Non-SGLT2i users) | Adjusted hazard ratio (95% CI)[a] | | | |
|---|---|---|---|---|
| | **1 day to 1y** | **1 day to 3y** | **1 day to 5y** | **1 day to anytime[b]** |
| Lupus nephritis | **0.44 (0.29-0.67)** | **0.55 (0.39-0.77)** | **0.55 (0.40-0.77)** | **0.61 (0.44-0.82)** |
| Dialysis | **0.17 (0.07-0.40)** | **0.27 (0.15-0.47)** | **0.29 (0.17-0.48)** | **0.36 (0.23-0.56)** |
| Kidney transplant | NA | **0.16 (0.04-0.72)** | **0.14 (0.03-0.62)** | **0.13 (0.03-0.58)** |
| Heart failure | **0.48 (0.36-0.63)** | **0.59 (0.48-0.74)** | **0.65 (0.53-0.78)** | **0.66 (0.55-0.79)** |
| All-cause mortality | **0.19 (0.11-0.33)** | **0.32 (0.23-0.45)** | **0.35 (0.26-0.47)** | **0.33 (0.25-0.44)** |

SGLT2i: Sodium-glucose co-transporter 2 inhibitors; CI: Confidence interval; NA: Not applicable.
[a] Propensity score matching was performed age at index, sex, race, social economic status, lifestyles, medical utilization, DM severity, angiotensin II receptor blockers (ARB), and estimated glomerular filtration rate (eGFR).
[b] Around 3000 days.
* Proportionality <0.001.

© 2024 Yen FS et al. *JAMA Network Open*.

**eTable 3. Risk of outcomes deals with the competing risk of death**

| Outcomes | Patients with outcome | | Adjusted hazard ratio (95% CI) [a] |
|---|---|---|---|
| | SGLT2i users (n=1775) | Non- SGLT2i users (n=1775) | |
| Lupus nephritis or deceased | 114 (6.42%) | 250 (14.08%) | **0.43 (0.35-0.54)** |
| Dialysis or deceased | 73 (4.11%) | 210 (11.83%) | **0.34 (0.26-0.44)** |
| Kidney transplant or deceased | 59 (3.32%) | 173 (9.75%) | **0.33 (0.25-0.45)** |
| Heart failure or deceased | 220 (12.39%) | 363 (20.45%) | **0.57 (0.48-0.68)** |
| All-cause mortality | 58 (3.27%) | 163 (9.18%) | **0.35 (0.26-0.47)** |

SGLT2i: Sodium-glucose co-transporter 2 inhibitors; CI: Confidence interval.
If the patient is less or equal to 10, results show the count as 10.
[a.] Propensity score matching was performed on age at index, sex, race, social economic status, lifestyles, medical utilization, DM severity, angiotensin II receptor blockers (ARB), and estimated glomerular filtration rate (eGFR).
* Proportionality <0.001.

© 2024 Yen FS et al. *JAMA Network Open*.

**eTable 4. Risk of outcomes stratified by HbA1C level (7%)**

| **Outcomes (SGLT2i users vs. Non-SGLT2i users)** | **Adjusted hazard ratio (95% CI)[a]** | |
|---|---|---|
| | **HbA1C < 7 %[b] (n= 174 pairs)** | **HbA1C ≧ 7 %[c] (n= 752 pairs)** |
| Lupus nephritis | 0.88 (0.34-2.28) | 0.94 (0.53-1.65) |
| Dialysis | 2.49 (0.48-12.86) | **0.21 (0.09-0.50)** |
| Kidney transplant | NA | NA |
| Heart failure | 0.66 (0.36-1.20) | **0.56 (0.41-0.76)** |
| All-cause mortality | **0.29 (0.12-0.73)** | **0.35 (0.22-0.56)** |

HbA1C: Hemoglobin A1c; SGLT2i: Sodium-glucose co-transporter 2 inhibitors; CI: Confidence interval; NA: Not applicable.
[a] Propensity score matching was performed on age at index, sex, race, social economic status, lifestyles, medical utilization, DM severity, angiotensin II receptor blockers (ARB), and estimated glomerular filtration rate (eGFR).
[b] Prior to the index date, the HbA1C results consistently remained below 7%, with no instances recorded as equal to or greater than 7% in their electronic medical records.
[c] Prior to the index date, subjects had HbA1C levels equal to or greater than 7% recorded in their electronic medical records.
* Proportionality <0.001.

© 2024 Yen FS et al. *JAMA Network Open*.

**eTable 5. Risk of outcomes stratified by HbA1C level (9%)**

| **Outcomes (SGLT2i users vs. Non-SGLT2i users)** | **Adjusted hazard ratio (95% CI)[a]** | |
|---|---|---|
| | **HbA1C < 9 %[b] (n=584 pairs)** | **HbA1C ≧ 9 %[c] (n=307 pairs)** |
| Lupus nephritis | 0.86 (0.44-1.68) | **0.33 (0.14-0.78)** |
| Dialysis | 0.48 (0.20-1.18) | **0.12 (0.03-0.52)** |
| Kidney transplant | NA | NA |
| Heart failure | **0.56 (0.40-0.78)** | **0.50 (0.31-0.82)*** |
| All-cause mortality | **0.20 (0.11-0.38)** | **0.38 (0.20-0.74)** |

HbA1C: Hemoglobin A1c; SGLT2i: Sodium-glucose co-transporter 2 GLT2) inhibitors; CI: Confidence interval; NA: Not applicable.

[a] Propensity score matching was performed on age at index, sex, race, social economic status, lifestyles, medical utilization, DM severity, angiotensin II receptor blockers (ARB), and estimated glomerular filtration rate (eGFR).

[b] Prior to the index date, the HbA1C results consistently remained below 9%, with no instances recorded as equal to or greater than 9% in their electronic medical records.

[c] Prior to the index date, subjects had HbA1C levels equal to or greater than 9% recorded in their electronic medical records.

* Proportionality <0.001.

© 2024 Yen FS et al. *JAMA Network Open*.

**eTable 6. Risk of outcomes stratified by creatinine level**

| Outcomes (SGLT2i users vs. Non-SGLT2i users) | Adjusted hazard ratio (95% CI)[a] | |
|---|---|---|
| | Creatinine < 1.5 mg/dL[b] (n=1202 pairs) | Creatinine ≧ 1.5 mg/dL[c] (n=141 pairs) |
| Lupus nephritis | 0.80 (0.49-1.29) | 0.69 (0.34-1.40) |
| Dialysis | 0.51 (0.25-1.04) | **0.17 (0.06-0.48)** |
| Kidney transplant | NA | NA |
| Heart failure | **0.73 (0.56-0.94)** | **0.51 (0.30-0.87)** |
| All-cause mortality | **0.36 (0.24-0.52)** | **0.37 (0.17-0.78)** |

SGLT2i: Sodium-glucose co-transporter 2 inhibitors; CI: Confidence interval; NA: Not applicable.
[a] Propensity score matching was performed on age at index, sex, race, social economic status, lifestyles, medical utilization, DM severity, angiotensin II receptor blockers (ARB), and estimated glomerular filtration rate (eGFR).
[b] Prior to the index date, the creatinine in serum consistently remained below 1.5 mg/dL, with no instances recorded as equal to or greater than 1.5 mg/dL in their electronic medical records.
[c] Prior to the index date, subjects had creatinine in serum equal to or greater than 1.5 mg/dL recorded in their electronic medical records.
* Proportionality <0.001.

© 2024 Yen FS et al. *JAMA Network Open*.

**eTable 7. Risk of outcomes stratified by eGFR**

| Outcomes (SGLT2i users vs. Non-SGLT2i users) | Adjusted hazard ratio (95% CI)[a] | |
|---|---|---|
| | **eGFR < 60 mL/min/1.73m$^2$ [b] (n=578 pairs)** | **eGFR ≧ 60 mL/min/1.73m$^2$ [c] (n=731 pairs)** |
| Lupus nephritis | 0.81 (0.49-1.34) | 0.62 (0.32-1.19) |
| Dialysis | **0.29 (0.12-0.56)** | 1.34 (0.42-4.21) |
| Kidney transplant | NA | NA |
| Heart failure | **0.51 (0.38-0.69)**[*] | **0.61 (0.43-0.87)** |
| All-cause mortality | **0.39 (0.25-0.61)** | **0.37 (0.22-0.63)** |

eGFR: estimate Glomerular filtration rate; SGLT2i: Sodium-glucose co-transporter 2 inhibitors; CI: Confidence interval; NA: Not applicable.

[a] Propensity score matching was performed on age at index, sex, race, social economic status, lifestyles, medical utilization, DM severity, angiotensin II receptor blockers (ARB), and estimated glomerular filtration rate (eGFR).

[b] Prior to the index date, subjects had the eGFR below 60 mL/min/1.73m$^2$ recorded in their electronic medical records.

[c] Prior to the index date, subjects had eGFR equal to or greater than 60 mL/min/1.73m$^2$, with no instances recorded as below 60 mL/min/1.73m$^2$ recorded in their electronic medical records.

* Proportionality <0.001.

© 2024 Yen FS et al. *JAMA Network Open*.

**eTable 8. Risk of outcomes stratified by CKD**

| **Outcomes (SGLT2i users vs. Non-SGLT2i users)** | **Adjusted hazard ratio (95% CI)[a]** | |
|---|---|---|
| | **With CKD [b] (n=171 pairs)** | **Without CKD [c] (n=1553 pairs)** |
| Lupus nephritis | 0.65 (0.33-1.27) | **0.58 (0.39-0.85)** |
| Dialysis | **0.20 (0.06-0.69)** | **0.43 (0.24-0.77)** |
| Kidney transplant | NA | 0.37 (0.07-1.93) |
| Heart failure | **0.56 (0.33-0.95)** | **0.68 (0.55-0.85)** |
| All-cause mortality | **0.30 (0.14-0.65)** | **0.41 (0.30-0.57)** |

CKD, Chronic Kidney Diseases; SGLT2i, Sodium-glucose co-transporter 2 inhibitors; CI, Confidence interval; NA, Not applicable.
[a] Propensity score matching was performed on age at index, sex, race, social economic status, lifestyles, medical utilization, DM severity, angiotensin II receptor blockers (ARB), and estimated glomerular filtration rate (eGFR).
[b] Prior to the index date, subjects had been diagnosed with CKD (ICD 10 code N18), as recorded in their electronic medical records.
[c] Prior to the index date, subjects had no record of comorbid CKD (ICD 10 code N18) in their electronic medical records.
* Proportionality <0.001.

© 2024 Yen FS et al. *JAMA Network Open*.

**eTable 9. Risk of outcomes stratified by kidney complications**

| Outcomes<br>(SGLT2i users vs. Non-SGLT2i users) | Adjusted hazard ratio (95% CI)[a] | |
|---|---|---|
| | With kidney complications [b]<br>(n=126 pairs) | Without kidney complications [c]<br>(n=1540 pairs) |
| Lupus nephritis | 0.80 (0.34-1.85) | **0.66 (0.44-0.97)** |
| Dialysis | 0.32 (0.09-1.16) | **0.45 (0.24-0.83)** |
| Kidney transplant | NA | **0.17 (0.04-0.78)** |
| Heart failure | **0.34 (0.17-0.65)** | **0.72 (0.58-0.90)** |
| All-cause mortality | 0.44 (0.18-1.08) | **0.44 (0.31-0.61)** |

SGLT2i, Sodium-glucose co-transporter 2 inhibitors; CI, Confidence interval; NA, Not applicable.

[a] Propensity score matching was performed on age at index, sex, race, social economic status, lifestyles, medical utilization, DM severity, angiotensin II receptor blockers (ARB), and estimated glomerular filtration rate (eGFR).

[b] Prior to the index date, subjects had been diagnosed with Type 2 diabetes mellitus with kidney complications (ICD 10 code E11.2), as recorded in their electronic medical records.

[c] Prior to the index date, subjects had no record of comorbid with kidney complications (ICD 10 code E11.2) in their electronic medical records.

* Proportionality <0.001.

© 2024 Yen FS et al. *JAMA Network Open*.

**eTable 10. Risk of outcomes between matched SGLT2i and sulfonylurea users in patients with systemic lupus erythematosus**

| Outcomes | Patients with outcome | | Adjusted hazard ratio (95% CI) [a] |
|---|---|---|---|
| | SGLT2i users (n=998) | Sulfonylureas users (n=998) | |
| Lupus nephritis | 31 (3.11%) | 49 (4.91%) | 0.74 (0.47-1.17) |
| Dialysis | 10 (1.00%) | 31 (3.11%) | **0.39 (0.19-0.79)** |
| Kidney transplant | 10 (1.00%) | 10 (1.00%) | 0.15 (0.02-1.17) |
| Heart failure | 95 (9.52%) | 135 (13.53%) | 0.81 (0.62-1.06) |
| All-cause mortality | 38 (3.81%) | 91 (9.12%) | **0.54 (0.37-0.78)** |

SGLT2i, Sodium-glucose co-transporter 2 inhibitors; CI, Confidence interval.
If the number of patients is fewer than or equal to 10, results show the count as 10.
[a.] Propensity score matching was performed on age at index, sex, race, social economic status, lifestyles, medical utilization, DM severity, angiotensin II receptor blockers (ARB), and estimated glomerular filtration rate (eGFR).
* Proportionality <0.001.

© 2024 Yen FS et al. *JAMA Network Open*.

**eTable 11. Risk of outcomes between matched SGLT2i and DPP-4i users in patients with systemic lupus erythematosus**

| Outcomes | Patients with outcome | | Adjusted hazard ratio (95% CI) [a] |
|---|---|---|---|
| | SGLT2i users (n=1004) | DPP-4i users (n=1004) | |
| Lupus nephritis | 29 (2.89%) | 32 (3.19%) | 1.09 (0.66-1.81) |
| Dialysis | 16 (1.59%) | 24 (2.39%) | 0.82 (0.43-1.54) |
| Kidney transplant | 10 (1.00%) | 10 (1.00%) | 0.65 (0.12-3.57) |
| Heart failure | 101 (10.06%) | 130 (12.95%) | 0.93 (0.72-1.21) |
| All-cause mortality | 35 (3.49%) | 97 (9.66%) | **0.45 (0.30-0.66)** |

SGLT2i, Sodium-glucose co-transporter 2 inhibitors; DPP-4i, dipeptidyl peptidase-4 inhibitors; CI, Confidence interval.
If the number of patients is fewer than or equal to 10, results show the count as 10.
[a.] Propensity score matching was performed on age at index, sex, race, social economic status, lifestyles, medical utilization, DM severity, angiotensin II receptor blockers (ARB), and estimated glomerular filtration rate (eGFR).
* Proportionality <0.001.

© 2024 Yen FS et al. *JAMA Network Open*.

**eTable 12. Risk of outcomes with at least 2 vs no SGLT2i prescriptions**

| Outcomes | Patients with outcome | | Adjusted hazard ratio (95% CI) [a] |
|---|---|---|---|
| | SGLT2i users (n=1080) | Non- SGLT2i users (n=1080) | |
| Lupus nephritis | 40 (3.70%) | 56 (5.19%) | **0.64 (0.42-0.95)** |
| Dialysis | 11 (1.02%) | 32 (2.96%) | **0.31 (0.16-0.62)** |
| Kidney transplant | 10 (0.93%) | 10 (0.93%) | **0.11 (0.01-0.90)** |
| Heart failure | 116 (10.74%) | 155 (14.35%) | **0.67 (0.52-0.85)** |
| All-cause mortality | 32 (2.96%) | 84 (7.78%) | **0.35 (0.23-0.52)** |

SGLT2i: Sodium-glucose co-transporter 2 inhibitors, CI: Confidence interval.
If the number of patients is fewer than or equal to 10, results show the count as 10.
[a.] Propensity score matching was performed on age at index, sex, race, social economic status, lifestyles, medical utilization, DM severity, angiotensin II receptor blockers (ARB), and estimated glomerular filtration rate (eGFR).
* Proportionality <0.001.

© 2024 Yen FS et al. *JAMA Network Open*.
