# Supplementary material for: Sodium-Glucose Cotransporter-2 Inhibitors and Nephritis Among Patients With Systemic Lupus Erythematosus
Source: JAMA Netw Open. 2024 Jun 12;7(6):e2416578. doi: 10.1001/jamanetworkopen.2024.16578 (PMC11170305; doi:10.1001/jamanetworkopen.2024.16578)
Supplement: Supplement 2. — Data Sharing Statement [file jamanetwopen-e2416578-s002.pdf]

Data Sharing Statement

Yen. Sodium-Glucose Cotransporter-2 Inhibitors and Nephritis Among Patients With Systemic Lupus Erythematosus. *JAMA Netw Open*. Published June 12, 2024. doi:10.1001/jamanetworkopen.2024.16578

Data
Data available: No

Additional Information
Explanation for why data not available: It only can be obtain on reasonable request. Further information about the data of TriNetX can be accessed on their website: https://trinetx.com/?mc_cid=7e2ecd5bc5&mc_eid=%5BUNIQID%5D .
